# Supplementary material for: Cryo-EM structure of a functional monomeric Photosystem I from Thermosynechococcus elongatus reveals red chlorophyll cluster
Source: Commun Biol. 2021 Mar 8;4:304. doi: 10.1038/s42003-021-01808-9 (PMC7940658; doi:10.1038/s42003-021-01808-9)
Supplement: Supplementary file 2 — Supplementary Information [file 42003_2021_1808_MOESM2_ESM.pdf]

Supplementary Information

Cryo-EM structure of a functional monomeric Photosystem I from *Thermosynechococcus elongatus* reveals 'red' chlorophyll cluster

Orkun Çoruh[¶,1,2], Anna Frank[¶,3], Hideaki Tanaka[1], Akihiro Kawamoto[1], Eithar El-Mohsnawy[4], Takayuki Kato[5], Keiichi Namba[6,7,8], Christoph Gerle[1]*, Marc M. Nowaczyk*[3], Genji Kurisu[1,2]*

1 Laboratory for Protein Crystallography, Institute for Protein Research, Osaka University, 3-2 Yamada Oka, Suita, Osaka 565-0871, Japan
2 Department of Macromolecular Science, Graduate School of Science, Osaka University, 1-1 Machikaneyama, Toyonaka, Osaka 560-0043, Japan
3 Plant Biochemistry, Faculty of Biology and Biotechnology, Ruhr-University Bochum, Universitätsstr. 150, 44780 Bochum, Germany
4 Department of Botany and Microbiology, Faculty of Science, Kafrelsheikh University, Mubarak Rd., Kafr Al Sheikh, Egypt
5 Laboratory of CryoEM Structural Biology, Institute for Protein Research, Osaka University, 3-2 Yamada Oka, Suita, Osaka 565-0871, Japan
6 Graduate School of Frontier Biosciences, Osaka University, 1-3 Yamadaoka, Suita, Osaka 565-0871, Japan
7 RIKEN Center for Biosystems Dynamics Research and SPring-8 Center, 1-3 Yamadaoka, Suita, Osaka 565-0871, Japan
8 JEOL YOKOGUSHI Research Alliance Laboratories, Osaka University, 1-3 Yamadaoka, Suita, Osaka 565-0871, Japan

[¶] These authors contributed equally.

* Correspondence to: gerle.christoph@protein.osaka-u.ac.jp, marc.m.nowaczyk@rub.de or gkurisu@protein.osaka-u.ac.jp

Supplementary Methods

Crystallization of PSI monomer from *T. elongatus*

PSI monomer crystals were obtained by hanging-drop vapor diffusion method at 277.15 K. A hanging drop was prepared by mixing equal volumes of protein solution (3 mg Chl/ml) and reservoir solution containing 50 mM Tris pH 7.5, 50 mM NaCl and 20% (w/v) Polyethylene glycol (PEG) 400. For X-ray intensity data collection, the single crystals were transferred to a cryo-protectant solution containing the same buffer of crystallization buffer with 30% (w/v) ethylen glycol and immediately frozen by plunge freezing in liquid nitrogen.

X-ray crystallography of the PSI monomer crystal

Native data was collected on beamline BL44XU at SPring-8 using CCD detector MX-300HE (Rayonix) at cryogenic temperature (100 K). The native data set was processed and scaled using program XDS[1] and diffraction data at 6.5 Å resolution with 98.46% completeness was obtained from 180 frames. A crystal of PSI monomer belonged to space group P 32 2 1 with cell dimensions of a=187.029 Å, b= 187.029 Å, c= 233.805 Å, α= 90°, β= 90°, γ= 120°. The crystal contained one molecule in an asymmetric unit. Initial phase was determined by molecular replacement method with program PHASER in CCP4[2] using the X-ray structure of PSI (PDB ID 1JB0) as a starting model. The initial electron density map revealed most of the main chains of PSI. The structure model was manually revised using program Coot[3] in CCP4, and external restraints refinement was performed using REFMAC5 and ProSMART[4] in CCP4. All figures showing the atomic coordinates were made with ChimeraX[5].

Supplementary Figures

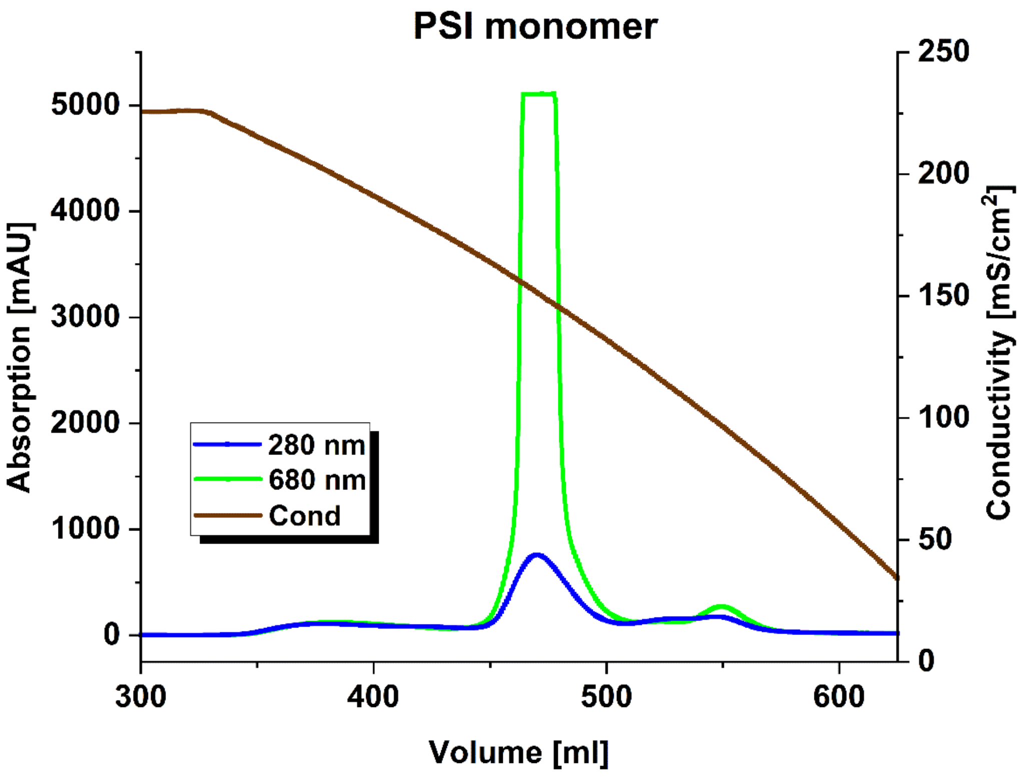

Supplementary Figure 1: HIC chromatography of monomeric PSI. HIC column chromatogram with the 680 nm absorption peak of the monomer fraction indicated.

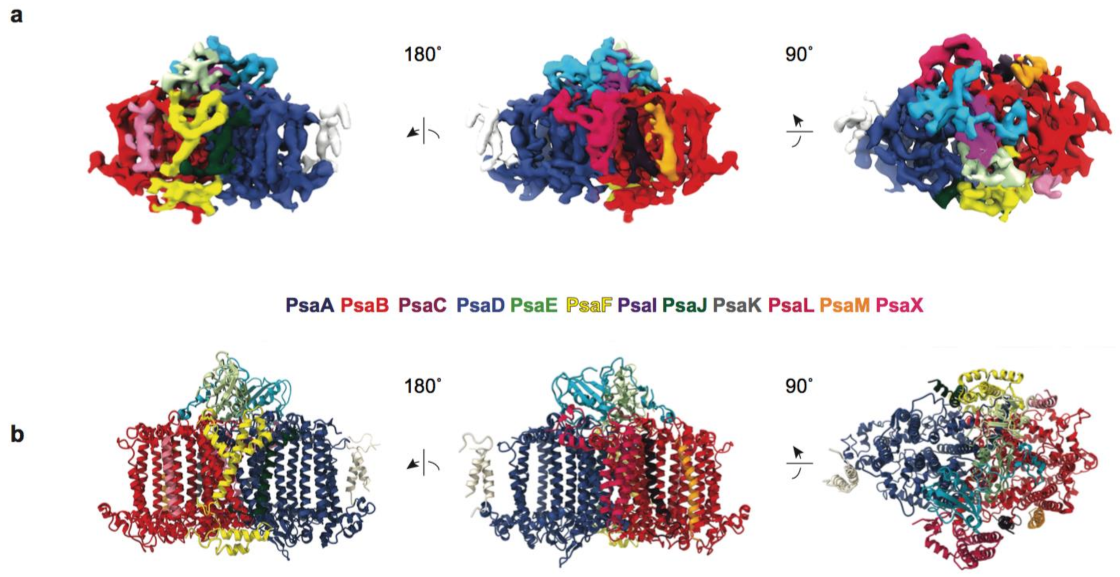

Supplementary Figure 2: The X-ray crystal structure model of the PSI monomer (PDB ID 7WB2) determined to 6.5 Å. View parallel to membrane from the membrane side (left), trimerization side (middle) and view along the membrane normal from the stromal side. Coloring of subunits as in Figure 1 and according to Jordan et al.[6], as indicated by the font color of the subunit names. **a** Calculated X-ray electron density map and **b** X-ray based model.

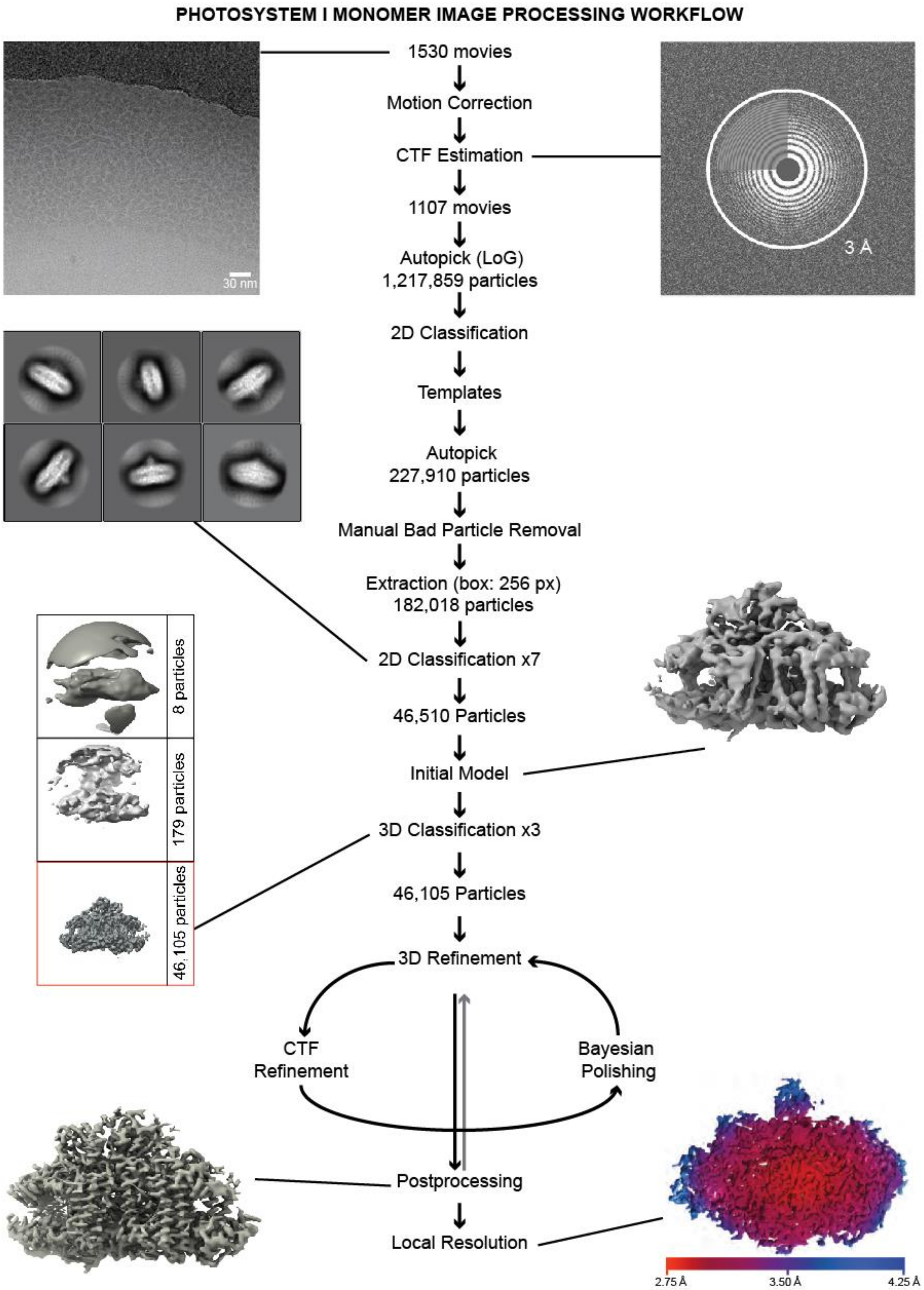

Supplementary Figure 3: Flowchart of single-particle cryo-EM image processing of the *T. elongatus* PSI monomer structure (PDB ID 6LU1, EMDB ID EMD-0977, EMPIAR-10352).

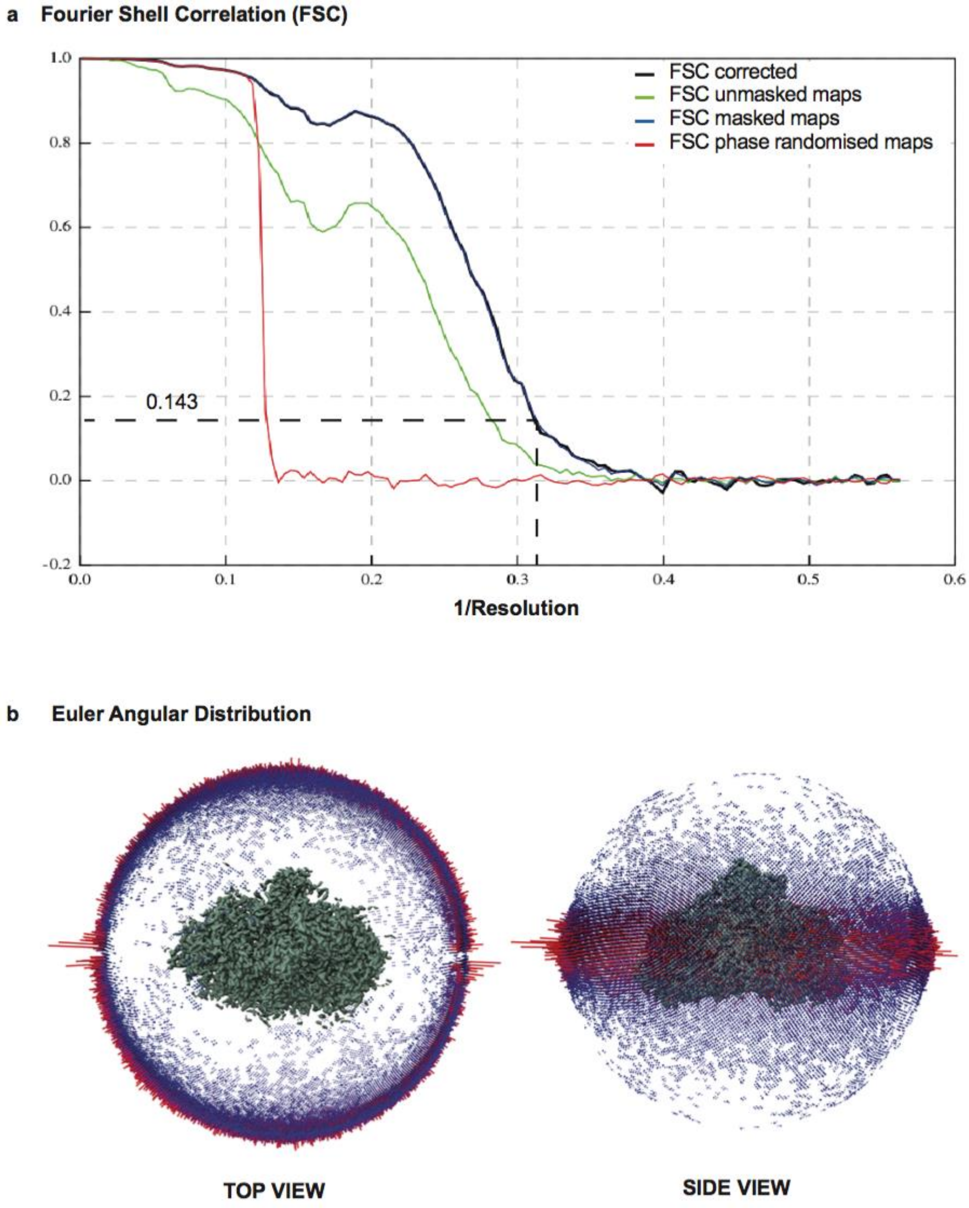

Supplementary Figure 4: Fourier Shell Correlation and Euler Angular Distribution a FSC curves **b** Euler angle distribution of the final single particle cryo-EM map (EMDB ID: EMD-0977)

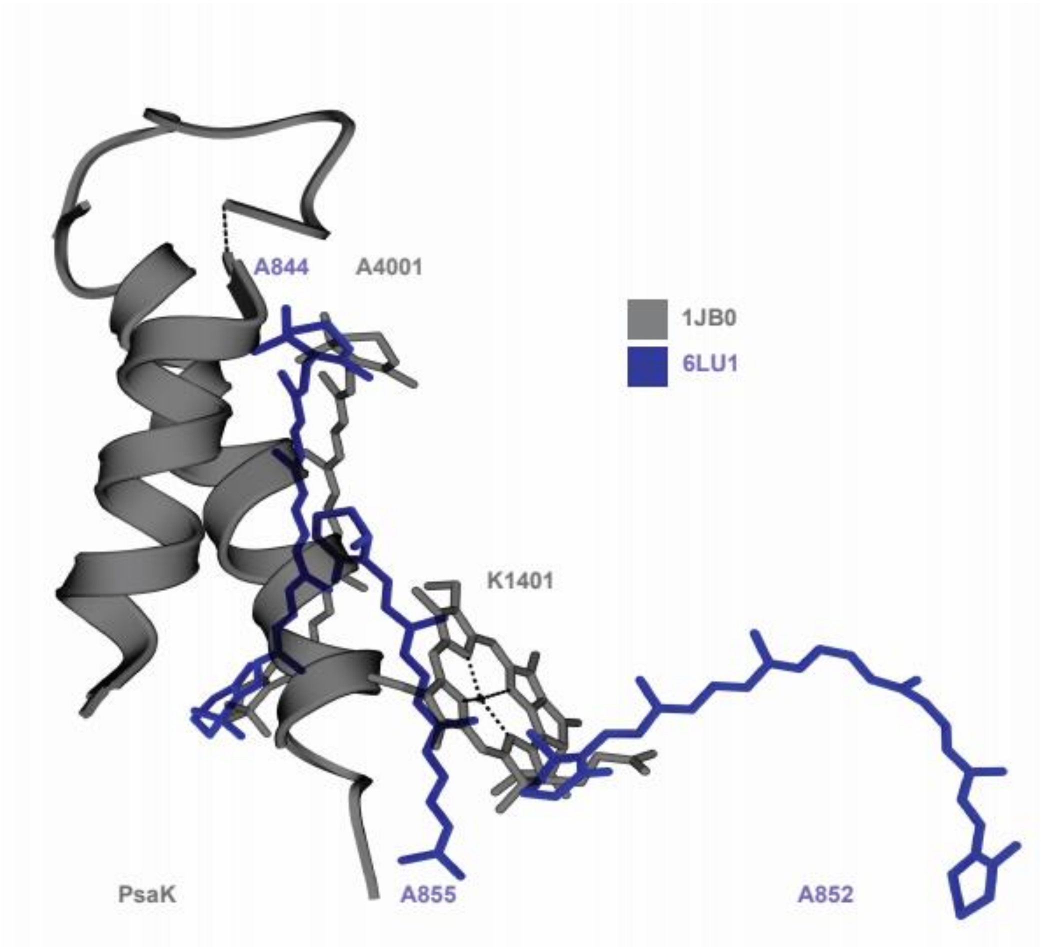

Supplementary Figure 5: Mobility of carotenoids upon monomerization. In the PSI monomer structure (PDB ID: 6LU1) newly found carotenoids A855 and A852 are clashing with the position of PsaK and its chlorophyll in the trimer structure. Likewise, PsaK-bound carotenoid A4001 of the trimer structure, named A844 in the monomer structure, shifts its position to a degree that it clashes with the space occupied by PsaK in the trimer structure.

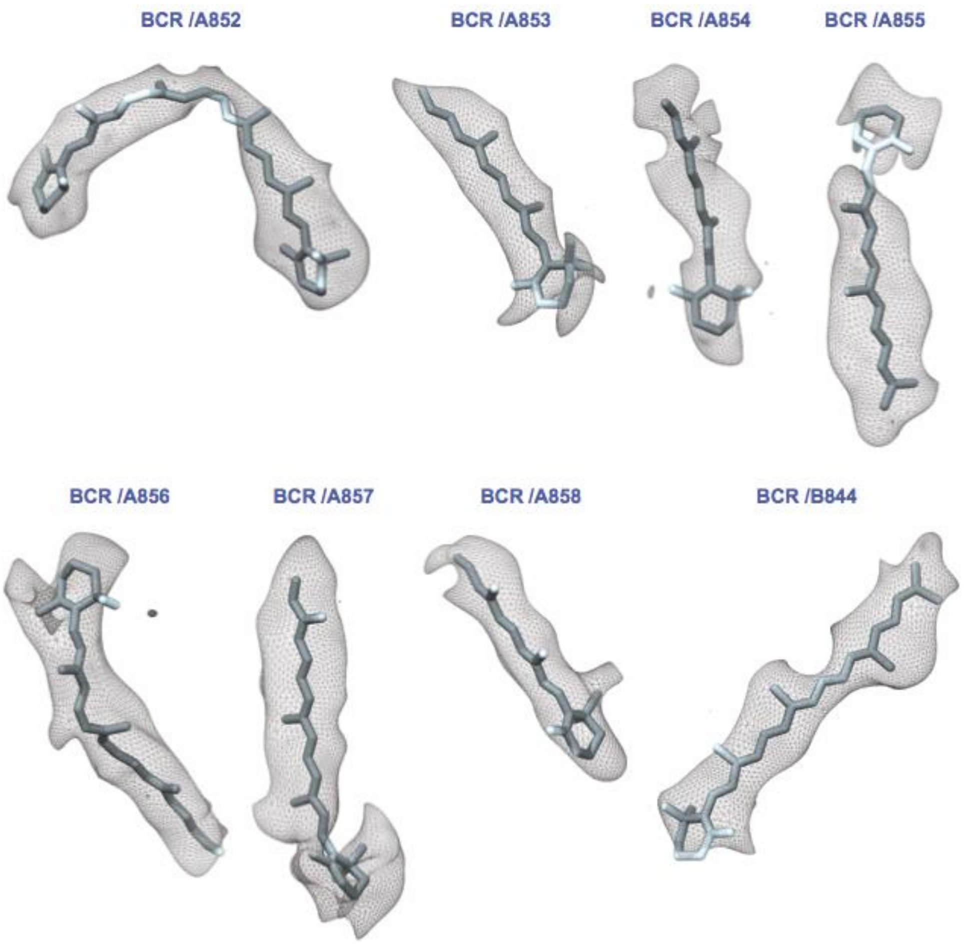

Supplementary Figure 6: Newly modelled carotenoids with corresponding density maps. The carotenoids are viewed in an orientation matching the sideview in Figure 6.

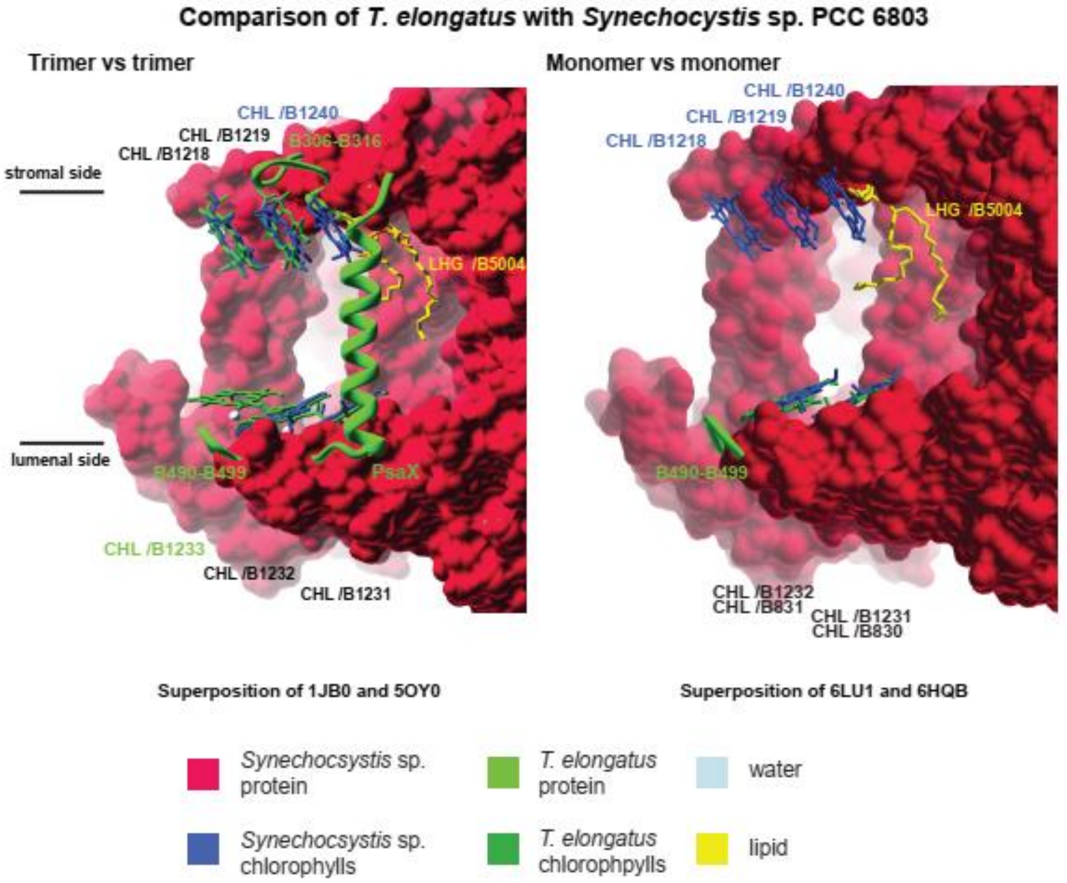

Structural differences between PSI from *T. elongatus* and *Synechocystis* sp. at the 'red' chlorophyll cluster region	
Between trimer structures	*Between monomer structures*
Stromal loop (B306-B316) above B1219 in *T. elongatus*	Disorder of B1218 and 1219 amongst other chlorophylls in *T. elongatus*
Chlorophyll B1233 to form a trimer with B1232 and B1231 in *T. elongatus*	Absence of B1240 in *T. elongatus*
Lumenal loop (B490-B499) below B1233 in *T. elongatus*	Presence of lipid B5004 in *Synechocystis* sp.
Chlorophyll B1240 forming a trimer with B1218 and B1219 in *Synechocystis* sp.	Absence of a loop (B490-B499) in *Synechocystis* sp.

Supplementary Figure 7: Structural differences between *T. elongatus*[6] and *Synechocystis* sp. PCC 6803[7,8] PSI in the 'red' chlorophyll cluster region on the periphery of PsaB.

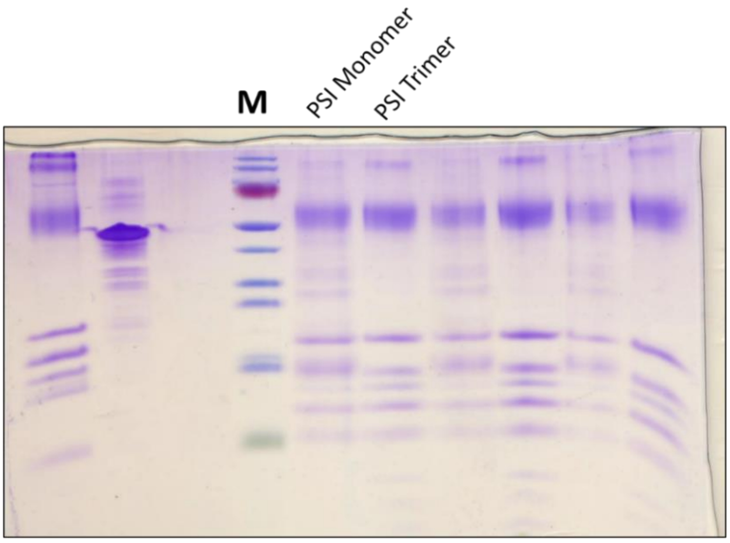

Supplementary Figure 8: Schägger-Jagow-PAGE of monomeric and trimeric PSI (Uncropped). Both samples from T. elongatus BP-1 WT, M: Protein Standard (PageRuler™ Prestained, Thermo Fischer Scientific Inc.), 2.5 µg Chl per sample.

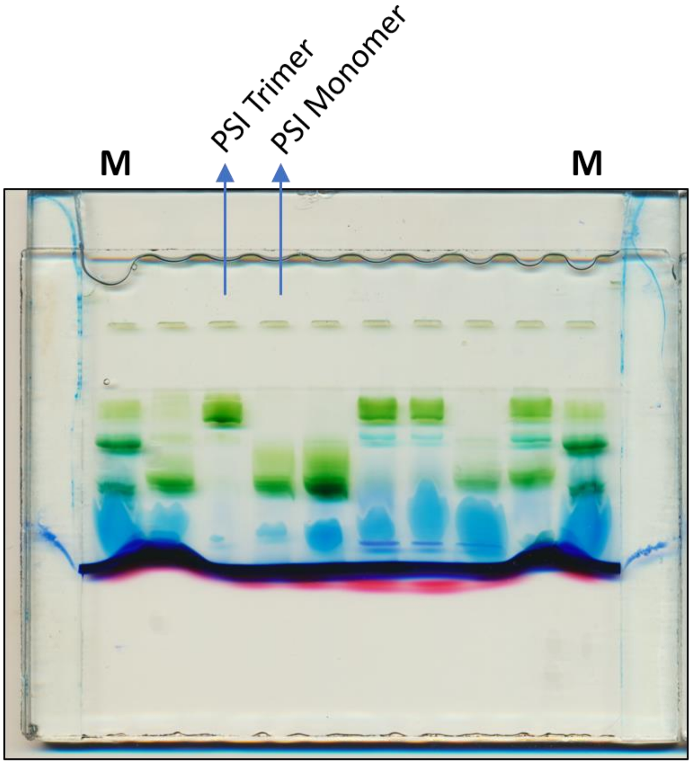

Supplementary Figure 9: BN-PAGE of monomeric and trimeric PSI from T. elongatus (Uncropped). M: Protein Standard (PSI Trimer (~1000 kDa), PSII Dimer (~500 kDa), PSII Monomer (~250 kDa)), 3 µg Chl per sample, trimeric PSI was isolated from T. elongatus BP-1 PsaE-his via immobilized metal ion affinity chromatography (IMAC), monomeric PSI was isolated from wildtype T. elongatus BP-1 via HIC.

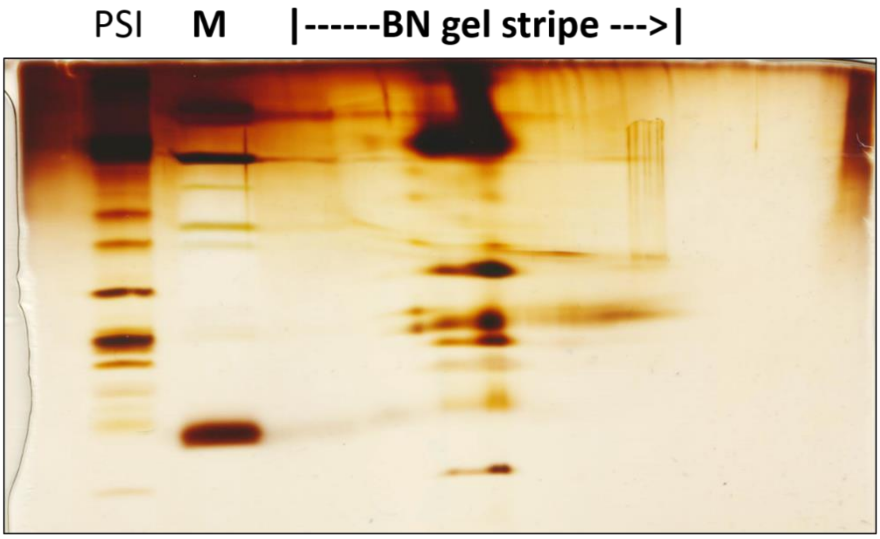

Supplementary Figure 10: 2D-PAGE of monomeric PSI from T. elongatus (Uncropped). The BN Gel stripe (PSI monomer, Figure SII) was stacked onto a denaturizing SJ-gel as a second dimension with the gradient increasing from left to right, PSI: PSI monomer standard (1.5 µg Chl), M: Protein Standard (PageRuler™ Unstained, Thermo Fischer Scientific Inc.).

Supplementary Tables

Supplementary Table 1: Percentage of modelled residues in 6LU1 and 1JB0[6].

SUBUNIT	6LU1 RESIDUES MODELLED (%)	1JB0 RESIDUES MODELLED (%)
PsaA	95	95
PsaB	95	100
PsaC	98	100
PsaD	99	100
PsaE	91	92
PsaF	18	86
PsaI	100	100
PsaJ	20	100
PsaK	0	55
PsaL	87	95
PsaM	100	100
PsaX	0	83

Supplementary Table 2: Q scores -a measure of resolvability of individual atoms- for individual subunits, modelled chlorophylls, modelled carotenoids and newly modelled carotenoids.

Chain	Q-score	Estimated Resolution (Å)
A	0.71	2.30
B	0.70	2.37
C	0.69	2.41
D	0.68	2.50
E	0.64	2.69
F	0.48	3.54
I	0.65	2.62
J	0.52	3.40
K	-	-
L	0.64	2.68
M	0.66	2.59
X	-	-
Chlorophyll *a*	0.71	-
Carotenoids	0.61	-
New Carotenoids	0.53	-

Supplementary Table 3: Comparison of distances in the ETC of PSI of X-ray and cryo-EM-based models analyzed to comparable resolution, using different refinement strategies. All distances were measured as in Jordan et al., 2001, that is from Mg atom to Mg atom between chlorophylls and between Mg atom and the center of the phylloquinone carbonyls and the center of iron-sulfur clusters.

	Çoruh et al. (2020)	Çoruh et al. (2020)	Jordan et al. (2001)[6]	Suga et al. (2019)[9]	Zheng et al. (2019)[10]	Netzer-El et.al. (2019)[7]
PDB-ID<br>METHOD<br>RESOLUTION	6LU1<br>Cryo-EM<br>3.2 Å<br>(2.7 Å**)	-*<br>Cryo-EM<br>3.2 Å<br>(2.7 Å**)	1JB0<br>X-Ray<br>2.5 Å	6JO5<br>Cryo-EM<br>2.9 Å<br>(2.5 Å**)	6K61<br>Cryo-EM<br>2.4 Å<br>(2.2 Å*)	6HQ1<br>X-Ray<br>4 Å
			Distance (Å)			
A1-B2	12.4	12.0	11.7	11.9	12.1	12.3
B1-A2	12.6	12.0	12.0	11.9	11.8	12.2
B2-A3	8.2	8.7	8.8	7.9	8.3	8.3
A2-B3	7.7	8.4	8.2	7.6	7.9	7.8
A3-Q_KA	10.0	10.3	8.6	9.0	9.3	9.5
B3-Q_KB	9.8	10.1	14.2	8.6	8.9	9.0
Q_KA-F_X	14.5	14.6	14.1	14.4	14.1	13.9
Q_KB-F_X	14.5	14.9	14.9	14.5	13.9	14.2
F_X-F_A	15.8	15.7	14.9	14.9	15.1	14.7
F_X-F_B	12.6	12.6	12.3	12.3	12.3	12.0

*A refinement of final model with CSD Restraints

**Highest local resolution observable in the deposited map.

Supplementary References

1) W. Kabsch, XDS. *Acta Crystallogr. D* **66**, 125–132 (2010).

2) Collaborative Computational Project 4, The CCP4 suite: Programs for protein crystallography. *Acta Crystallogr. D* **50**, 760–763 (1994).

3) P. Emsley and K. Cowtan, Coot: Model-building tools for molecular graphics. *Acta Crystallogr. D* **60**, 2126–2132 (2004).

4) R. A. Nicholls, F. Long and G. N. Murshudov, Low Resolution Refinement Tools in REFMAC5. *Acta Cryst. D* **68**. 404-417 (2012).

5) Goddard, T. D. et al. UCSF ChimeraX: Meeting modern challenges in visualization and analysis. Protein Sci. 27, 14–25 (2018).

6) Jordan, P. et al. Three-dimensional structure of cyanobacterial photosystem I at 2.5 Å resolution. Nature 411, 909-917 (2001). doi:10.1038/35082000.

7) Netzer-El, S. Y., Caspy, I. & Nelson, N. Crystal structure of photosystem I monomer from Synechocystis PCC 6803. Front. Plant Sci. 9, (2019).

8) Malavath, T., Caspy, I., Netzer-El, S. Y., Klaiman, D. & Nelson, N. Structure and function of wild-type and subunit-depleted photosystem I in Synechocystis. Biochim. Biophys. Acta - Bioenerg. 1859, 645–654 (2018).

9) Suga, Michihiro, Shin-Ichiro Ozawa, Kaori Yoshida-Motomura, Fusamichi Akita, Naoyuki Miyazaki, and Yuichiro Takahashi. "Structure of the green algal photosystem I supercomplex with a decameric light-harvesting complex I." *Nature Plants* **5** (6), 626-636 (2019).

10) Zheng, L. *et al.* Structural and functional insights into the tetrameric photosystem I from heterocyst-forming cyanobacteria. *Nat. Plants* **14**, 331-332 (2019). doi:10.1038/s41477-019-0525-6.
