# Supplementary material for: Cryo-EM structure of a functional monomeric Photosystem I from Thermosynechococcus elongatus reveals red chlorophyll cluster
Source: Commun Biol. 2021 Mar 8;4:304. doi: 10.1038/s42003-021-01808-9 (PMC7940658; doi:10.1038/s42003-021-01808-9)
Supplement: Supplementary file 3 — Description of Additional Supplementary Files [file 42003_2021_1808_MOESM3_ESM.pdf]

Description of Additional Supplementary Files

File Name: Supplementary Movie 1
Description: Single particle cryo-EM Coulomb map of the T. elongatus BP-1 PSI monomer (EMDB-0977) including the visualization of its detergent belt, the built atomic model (PDB-ID: 6LU1) and a comparison with the trimer crystal structure based model (PDB-ID: 1JB0).

File Name: Supplementary Data 1
Description: Raw data of UV-Visible absorption spectroscopy measurements.

File Name: Supplementary Data 2
Description: Raw data of 77 K fluorescence spectroscopy measurements.
