# Supplementary material for: Cryo-EM structure of a functional monomeric Photosystem I from Thermosynechococcus elongatus reveals red chlorophyll cluster
Source: Commun Biol. 2021 Mar 8;4:304. doi: 10.1038/s42003-021-01808-9 (PMC7940658; doi:10.1038/s42003-021-01808-9)
Supplement: Supplementary file 7 — Reporting Summary [file 42003_2021_1808_MOESM7_ESM.pdf]

nature research

Corresponding author(s): Christoph Gerle
Marc Nowacyzk
Genji Kurisu

Last updated by author(s): Dec 28, 2020

Reporting Summary

Nature Research wishes to improve the reproducibility of the work that we publish. This form provides structure for consistency and transparency in reporting. For further information on Nature Research policies, see our Editorial Policies and the Editorial Policy Checklist.

Statistics

For all statistical analyses, confirm that the following items are present in the figure legend, table legend, main text, or Methods section.

n/a	Confirmed	
☒	☐	The exact sample size (*n*) for each experimental group/condition, given as a discrete number and unit of measurement
☒	☐	A statement on whether measurements were taken from distinct samples or whether the same sample was measured repeatedly
☒	☐	The statistical test(s) used AND whether they are one- or two-sided<br>*Only common tests should be described solely by name; describe more complex techniques in the Methods section.*
☒	☐	A description of all covariates tested
☒	☐	A description of any assumptions or corrections, such as tests of normality and adjustment for multiple comparisons
☒	☐	A full description of the statistical parameters including central tendency (e.g. means) or other basic estimates (e.g. regression coefficient) AND variation (e.g. standard deviation) or associated estimates of uncertainty (e.g. confidence intervals)
☒	☐	For null hypothesis testing, the test statistic (e.g. *F*, *t*, *r*) with confidence intervals, effect sizes, degrees of freedom and *P* value noted<br>*Give P values as exact values whenever suitable.*
☒	☐	For Bayesian analysis, information on the choice of priors and Markov chain Monte Carlo settings
☒	☐	For hierarchical and complex designs, identification of the appropriate level for tests and full reporting of outcomes
☒	☐	Estimates of effect sizes (e.g. Cohen's *d*, Pearson's *r*), indicating how they were calculated

Our web collection on statistics for biologists contains articles on many of the points above.

Software and code

Policy information about availability of computer code

Data collection: Jadas Software : Automated Image Acquisition

Data analysis:
Relion 3.0 : Particle picking, 2D/3D classifications, particle alignment, 3D refinement, mask calculation and map sharpening
CryoSPARK : Particle picking, 2D/3D classifications, particle alignment, 3D refinement, mask calculation and map sharpening
MotionCor2 : Beam induced motion and radiation exposure related corrections
CTFFIND4 : Micrograph and particle contrast transfer function parameters calculation
Resmap : Local resolution estimation
MapQ : Resolvability estimation
Coot : Manual model building, refinement and validation, distance calculations
Phenix : Real space refinement with restraints and validation
UCSF Chimera : Mask and image preparation, visualization
USCF ChimeraX : Image preparation and visualization
XDS Native X-ray dataset scaling and processing
PHASER : Phase determination by molecular replacement
CCP4 : X-ray data analysis
Refmac : Restrained refinement
ProSMART : Restrained refinement

For manuscripts utilizing custom algorithms or software that are central to the research but not yet described in published literature, software must be made available to editors and reviewers. We strongly encourage code deposition in a community repository (e.g. GitHub). See the Nature Research guidelines for submitting code & software for further information.

Data

Policy information about availability of data

All manuscripts must include a data availability statement. This statement should provide the following information, where applicable:

- Accession codes, unique identifiers, or web links for publicly available datasets
- A list of figures that have associated raw data
- A description of any restrictions on data availability

Raw cryo-EM images of the T. elongatus PSI monomer after motion correction were deposited in the Electron Microscopy Public Image Archive (EMPIAR), under the accession number EMPIAR-10352. The cryo-EM density map of the T. elongatus PSI monomer has been deposited in the Electron Microscopy Data Bank (EMDB), under the accession number EMD-0977. The atomic coordinates of the T. elongatus PSI monomer cryo-EM structure and X-ray crystal structure have been deposited in the Protein Data Bank (PDB), under the accession number 6LU1 and 7WB2 respectively.

Field-specific reporting

Please select the one below that is the best fit for your research. If you are not sure, read the appropriate sections before making your selection.

☒ Life sciences ☐ Behavioural & social sciences ☐ Ecological, evolutionary & environmental sciences

For a reference copy of the document with all sections, see nature.com/documents/nr-reporting-summary-flat.pdf

Life sciences study design

All studies must disclose on these points even when the disclosure is negative.

Sample size	The final data set contains 46,105 independent particles
Data exclusions	Following the conventional practices of cryo-EM data processing, a significant amount of particles with high s/n ratio were excluded and this is clearly reported in methods section.
Replication	Final models produced rely on separate datasets produced from different experiments.
Randomization	As mentioned in the methods section, Relion software uses randomly splitting dataset into two groups and correlating it to estimate the final resolution as shown in FSC curve, provided in Supplementary Figure 4.
Blinding	The investigators were not blinded in any ways to identify the sample.

Reporting for specific materials, systems and methods

We require information from authors about some types of materials, experimental systems and methods used in many studies. Here, indicate whether each material, system or method listed is relevant to your study. If you are not sure if a list item applies to your research, read the appropriate section before selecting a response.

Materials & experimental systems

n/a	Involved in the study	
☒	☐	Antibodies
☒	☐	Eukaryotic cell lines
☒	☐	Palaeontology and archaeology
☒	☐	Animals and other organisms
☒	☐	Human research participants
☒	☐	Clinical data
☒	☐	Dual use research of concern

Methods

n/a	Involved in the study	
☒	☐	ChIP-seq
☒	☐	Flow cytometry
☒	☐	MRI-based neuroimaging
